# Extension of the ‘Inorganic Gel Casting’ Process to the Manufacturing of Boro-Alumino-Silicate Glass Foams

**DOI:** 10.3390/ma11122545

**Published:** 2018-12-14

**Authors:** Acacio Rincon Romero, Sergio Tamburini, Gianmarco Taveri, Jaromír Toušek, Ivo Dlouhy, Enrico Bernardo

**Affiliations:** 1 Department of Industrial Engineering, University of Padova, Via Marzolo 9, 35131 Padova, Italy; acacio.rinconromero@unipd.it; 2 National Research Council of Italy (CNR), Institute of Condensed Matter Chemistry and Technologies for Energy (ICMATE), Corso Stati Uniti 4, 35127 Padova, Italy; sergio.tamburini@cnr.it; 3 Institute of Physics of Materials (IPM), Žižkova 22, 61662 Brno, Czech Republic; taveri@drs.ipm.cz (G.T.); idlouhy@ipm.cz (I.D.); 4 CEITEC—Central European Institute of Technology, Masaryk University, Kamenice 5, 62500 Brno, Czech Republic; jaromir.tousek@ceitec.muni.cz

**Keywords:** glass recycling, alkali activation, gel casting, glass foams

## Abstract

A new technique for the production of glass foams, based on alkali activation and gel casting, previously applied to soda-lime glass, was successfully extended to boro-alumino-silicate glass, recovered from the recycling of pharmaceutical vials. A weak alkali activation (2.5 M NaOH or NaOH/KOH aqueous solutions) of fine glass powders (below 70 µm) allowed for the obtainment of well-dispersed concentrated aqueous suspensions, undergoing gelation by treatment at low temperature (75 °C). Unlike soda-lime glass, the progressive hardening could not be attributed to the formation of calcium-rich silicate hydrates. The gelation was provided considering the chemical formulation of pharmaceutical glass (CaO-free) to the formation of hydrated sodium alumino-silicate (N-A-S-H) gel. An extensive direct foaming was achieved by vigorous mechanical stirring of partially gelified suspensions, comprising also a surfactant. A sintering treatment at 700 °C, was finally applied to stabilize the cellular structures.

## 1. Introduction

The recovery of glass from differentiated urban waste collection is undoubtedly favourable, for the significant savings in raw materials and energy consumption upon melting (pre-formed glass act as a flux for the reaction of mineral raw materials) [1] but it must face important limitations. In fact, an ‘ideal’ recycling, corresponding to a complete reuse of glass cullet in the manufacturing of the original glass articles, technically known as ‘closed loop recycling’, is far from being feasible. It was estimated that the saving in embodied energy (energy to be committed to create a mass of usable material) using recycled material instead of ‘virgin’ raw materials is about 20% for glass, whereas it approaches 90% for aluminium [2].

The controversy of glass recycling basically concerns the quality of glass articles which may be significantly degraded, when employing not properly purified glass cullet [3,4]. Crushed glass, from municipal waste collection, is typically subjected to an expensive and difficult sorting step, aimed at separating glass pieces with different colours and removing metal, plastic or ceramic impurities, before being considered as a real alternative to minerals. Glass fractions, in which these impurities are concentrated, are still landfilled [5]. Even in the case of limited impurities, some glass may be discarded, if the original glass articles are no longer produced, as in the case of glasses from dismantled cathode ray tubes (TV and PC screens abandoned CRT technology more than a decade ago) [6]. Additional difficulties arise with glass articles deriving from a quite complex processing chain, like pharmaceutical vials: highly chemically stable boro-alumino-silicate containers are typically manufactured by thermo-mechanical processing of an intermediate product (glass tube), produced elsewhere [7].

The difficulties in the direct recycling of glass open the way to ‘open loop recycling’ applications, when glass is considered as a raw material for articles different from the original ones [6]. In this context, a fundamental challenge concerns the value of the new articles: the use of glass as simple sintering aid, in limited quantities, in several types of ceramics, although successful (waste-derived ceramics actually benefit from the chemical stability of pharmaceutical glass [6]), may configure just a ‘down-cycling’ option. Products maximizing the content of recycled glass, placed in an advantageous market ‘niche’, like glass foams [5,6], may configure, on the contrary, as an ‘up-cycling’ option, a truly profitable way of reusing discarded materials. 

The value of glass foams (or cellular glasses) is expressed by the distinctive set of properties, since they exhibit high strength-to-density ratios, high surface area, high permeability, low specific heat, high thermal and acoustic insulation combined with high chemical and thermal resistance (e.g., unlike polymer foams, glass foams are non-flammable and flame resistant) [8]. The value could be even maximized by optimization of the manufacturing process: in fact, while an energy-demanding melting process is avoided, exploiting the viscous flow sintering of glass powders, at much lower temperatures, the cost and the environmental impact of additives (‘foaming agents’), aimed at releasing gas (by oxidation or decomposition reactions) during sintering, are still disputable [9].

A substantial change in the approach to glass foams is offered by the separation of foaming and sintering steps, obtainable by gel casting technology [10], that may be applied to solutions (from sol-gel processing) [11], as well as to suspensions of glass powders [12,13]. Air bubbles are incorporated by intensive mechanical stirring, with the help of surfactants, forming a cellular structure, stabilized firstly by the progressive hardening (‘gelation’) of the starting slurry and secondly by the sintering treatment. Unlike conventional glass foams, foams from gel casting may keep the open-celled morphology achieved in the foaming step, of fundamental importance in high value applications, such as bone tissue engineering [12,13].

Recent investigations were focused at adjusting the gel casting technology to foams from recycled glasses in an ‘up-cycling’ perspective. Instead of deriving from expensive organic polymerization (due to the addition of monomers, cross-linkers and catalysts), used for biomaterials [12,13] gelation of glass slurries was achieved simply by alkali activation. As shown by Rincon et al. [5] for soda-lime glass, glass slurries in alkaline media (KOH aqueous solutions) exhibited a marked pseudoplastic behaviour, due to the formation of C-S-H (calcium silicate hydrated) gel at the surface of glass particles. At low shear rate, the gel could ‘glue’ the particles, trapping many air bubbles (incorporated upon intensive mechanical stirring, at high shear rate), at low temperature. Sintering could be performed at 700–800 °C, far below the temperatures used for soda-lime glass-based foams, produced with foaming agents.

The proposed ‘inorganic gel casting’ process was successfully applied to other glasses, also for the obtainment of glass-ceramic foams [14,15,16]. More precisely, the alkali activation of glass powders was followed by sinter-crystallization, consisting of viscous flow sintering of glass with concurrent crystallization. The precipitation of crystals, by enhancing the apparent viscosity of the glass mass during sintering, was significant in ‘freezing’ the open-celled structure, developed at low temperature. Soda-lime glass, on the contrary, experienced a ‘reshaping’ of pores, owing to the decomposition of the same hydrated compounds [5]. In all cases, the hardening could be attributed to the formation of a C-S-H gel (clearly visible by FTIR spectroscopy).

The investigation here presented was essentially aimed at extending the inorganic gel casting approach to glass foams with the above mentioned boro-alumino-silicate pharmaceutical glass known to feature a very limited CaO content and thus expected to lead to different gels [17]. In particular, the alkali activation was expected to lead, instead of a ‘tobermorite-like’ (C-S-H) gel, a truly ‘zeolite-like’ gel, determined by the bridging of [SiO_4_] and [AlO_4_] tetrahedra, in analogy with the usual alkali-activated materials generally known as ‘geopolymers’ [18]. The significant content of boron oxide was reputed as favourable, since it is well known to contribute to ‘zeolite-like’ networks in the form of [BO_4_] units [19]. Although still of not completely clear origin, the gelation effectively occurred with the manufacturing of open-celled glass foams possessing good strength-to-density ratios and a good ‘tunability’ of the microstructure according to the processing conditions.

## 2. Materials and Methods 

Pharmaceutical boro-alumino-silicated glass (later referred to as ‘BSG’; chemical composition [17]: SiO_2_ = 72 wt %, Al_2_O_3_ = 7 wt %, B_2_O_3_ = 12 wt %, Na_2_O = 6 wt %, K_2_O = 2 wt %, CaO = 1 wt %, BaO < 0.1 wt %) from crushed discarded vials, provided by the company Stevanato Group (Piombino Dese, Padova, Italy), was used as starting material.

Fine powders, after preliminary dry ball milling (Pulverisette 7 planetary ball mill, Fritsch, Idar-Oberstein, Germany) and manual sieving (<75 µm), were cast in aqueous solutions containing 2.5 M NaOH or 2.5 M NaOH/KOH (reagent grade, Sigma-Aldrich, Gillingham, UK) for a solid loading of 68 wt %. The glass powders were subjected to alkaline attack for 4 h under low speed mechanical stirring (500 rpm). After alkaline activation, the obtained suspensions of partially dissolved glass powders were poured in closed polystyrene cylindrical moulds (60 mm diameter) and dried at 75 °C for 4 h.

Partially dried suspensions were added with 4 wt % Triton X-100 (polyoxyethylene octyl phenyl ether—C_14_H_22_O(C_2_H_4_O)*_n_*, *n* = 9–10, Sigma-Aldrich, Gillingham, UK) and then foamed by vigorous mechanical mixing (2000 rpm). ‘Green’ foams were demoulded after a second drying step (at 75 °C, for 24 h) and fired at 700 °C for 1 h with a heating rate of 10 °C/min. For comparison purposes, some samples were produced by replacing Triton X-100 with Tween 80, a polyoxyethylene sorbitan monooleate—C_64_H_124_O_26_ (VWR BDH Prolabo, Milan, Italy) or an inexpensive ionic surfactant sodium lauryl sulphate (SLS; CH_3_(CH_2_)_11_OSO_3_Na (Carlo Erba, Cornaredo, Milan, Italy) in an aqueous solution 1/10 in weight.

Selected samples, in the hardened and in the fired state, were subjected to Fourier-transform infrared spectroscopy (FTIR, FTIR model 2000, Perkin Elmer Waltham, MA, USA), nuclear magnetic resonance (NMR) spectroscopy and X-ray diffraction (XRD) analysis. NMR analysis consisted of ^29^Si, ^27^Al and ^11^B studies: ^29^Si and ^27^Al spectra were collected on a Bruker AVANCE III spectrometer 300 (Bruker, Karlsruhe, Germany, magnetic field of 7.0 T corresponding to ^29^Si and ^27^Al Larmor frequencies of 59.623 and 78.066 MHz respectively) equipped for solid-state analysis in 4 mm diameter zirconia rotors. The magic angle was accurately adjusted prior to data acquisition using KBr. ^29^Si chemical shifts were externally referenced to solid tetrakis(trimetylsilyl)silane at –9.8 ppm (in relation to TMS) and ^27^Al chemical shifts were externally referenced to AlCl_3_·6H_2_O (0 ppm). The quantitative ^29^Si single-pulse experiments were collected at a spinning frequency of 6 kHz, a recycling delay of 100 s and 2000 transients. ^27^Al experiments were collected at a spinning frequency of 13 kHz with a recycle time of 2 s. About 4000 scans were needed using a single pulse experiment. 

^11^B NMR spectra were obtained using a Bruker Avance-500 spectrometer (Bruker, Karlsruhe, Germany), magnetic field of 11.7 T corresponding to ^11^B, Larmor frequencies of 160.462 MHz equipped for solid-state analysis in 4 mm diameter zirconia rotors. ^11^B chemical shifts were externally referenced to B(OAc)_3_ (3.0 ppm). The quantitative single-pulse experiments were collected at a spinning frequency of 14 kHz, a recycling delay of 5 s and 400 transients. 

The mineralogical analysis was conducted on powdered samples by means of X-ray diffraction (XRD) (Bruker D8 Advance, Karlsruhe, Germany), using CuKα radiation, 40 kV, 40 mA, 2θ = 10–70°, a step size of 0.02° and a counting time of 2 s, with a position sensitive detector (LinxEye, Bruker AXS, Karlsruhe, Germany). The phase identification was completed using the Match! programme package (Version 1.11, Crystal Impact GbR, Bonn, Germany), supported by data from the PDF-2 database (ICDD-International Centre for Diffraction Data, Newtown Square, PA, USA).

The morphological and microstructural characterizations were performed by scanning electron microscopy (FEI Quanta 200 ESEM, Eindhoven, The Netherlands).

The geometric density of both hardened foamed gels and fired glass foams was evaluated by considering the mass to volume ratio. The apparent and the true density were measured by using a helium pycnometer (Micromeritics AccuPyc 1330, Norcross, GA, USA), operating on bulk or on finely crushed samples, respectively. The three density values were used to compute the amounts of open and closed porosity.

Finally selected foams were subjected to compression and bending tests, employing samples of about 10 × 10 × 10 mm^3^ and of about 43 × 4 × 3 mm^3^, respectively, cut from larger specimens. Compression tests were applied by using an Instron 1121 UTS (Danvers, MA, USA) machine, with a crosshead speed of 1 mm/min (each data point corresponding to 10–12 samples). Bending tests were done on 3 × 4 mm^2^ specimens section by using Zwick/Roell Z50 machine (Ulm, Germany), operating in 3–4 point configuration (span = 16 mm and 40 mm, respectively), with a crosshead speed of 10 µm/min (each data point corresponding to 10 samples). 

## 3. Results and Discussion

**Figure 1:**
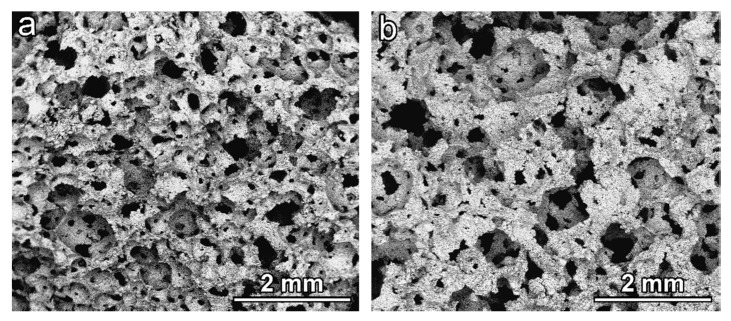
Morphology of foams in the ‘green’ state (before firing): (**a**) NaOH/KOH activation; (**b**) KOH activation.

As previously mentioned, the alkali activation determined the gelation of glass suspensions exploited for low temperature foaming, shown in Figure 1.

**Figure 2:**
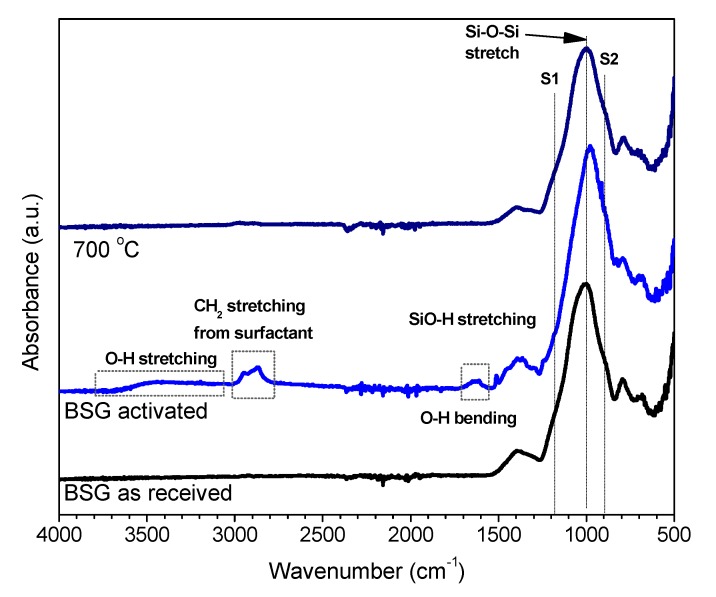
Fourier transform infrared (FTIR) analysis of boro-alumino-silicated glass (BSG) in the as received state, after alkali activation (in ‘green’ foams, NaOH/KOH activation) and after firing (in final foams)

FTIR spectroscopy was considered as the first step in investigating the nature of the developed gel. Compared to the patterns for the as received glass and for the foam after firing at 700 °C, the pattern for alkali activated BSG features limited differences, as illustrated by Figure 2 (although similar in behaviour, we report data only for the foams produced with an activating solution of 2.5 M NaOH/KOH, exhibiting the clearest FTIR pattern). In all spectra, the most intense band is centred around 1000–1050 cm^−1^ corresponding to tetrahedral stretching modes of two types of bridges, such as Si–O (Si) and Si–O (Al) [20]. The main band is accompanied by two broad shoulders, one at around 1200 cm^−1^ (S1), attributed to asymmetric stretching of B–O bonds in trigonal BO_3_, the other at around 900 cm^−1^ (S2), assigned to stretching vibrations of B–O bonds in BO_4_ tetrahedral unit [21,22]. 

The alkali activation, determining some broadening and downshifting of the main band, is interpreted as effects of the formation of a gel with mixed ions (in geopolymers, the band displacement is correlated with an increase of Al or B in the network structure [23]). The band at 900 cm^−1^, ascribable to the amount of boron in tetragonal configuration, visibly increased in intensity passing from the as received state to the activated and fired states. The bands around 800 and 1400 cm^−1^, attributed to the B–O bond bending vibration and asymmetric stretching vibration of the BO_3_ trigonal units, respectively [24,25,26], slightly decreased in intensity in the hardened foam.

Exclusively in the hardened foams, some additional bands were ascribed to the formation of silanol groups (SiOH) and incorporation of molecular water (SiO-H stretching at around 1600 cm^−1^, bending vibration of H_2_O at 1650 cm^−1^, to stretching vibration of H_2_O at 3430 cm^−1^ [21]). Finally, the band at about 2800 cm^−1^ was attributed to C-H_2_ stretch vibrations from the surfactant (undergoing complete decomposition upon heating) [5].

**Figure 3:**
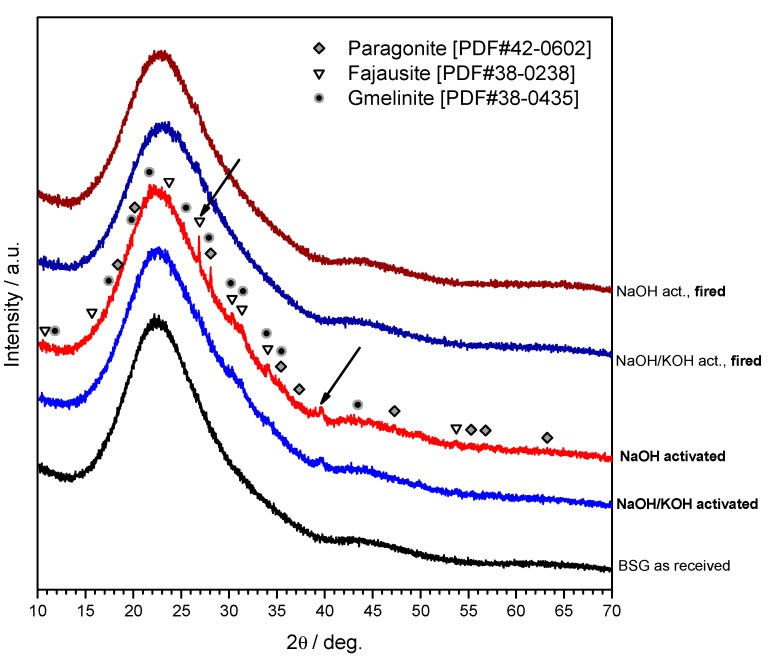
Mineralogical analysis of the studied materials.

The X-ray diffraction analysis (although performed with a position sensitive detector, yielding a distinctive high signal-to-noise ratio), shown in Figure 3, clarified only partially the gel nature. Considering that the patterns are nearly identical independently from the state (as received, hardened and fired), we can conclude that the gel was mostly amorphous and in limited quantities, unlike the case of soda-lime glass and other CaO rich glasses [5,14,15,16], for which the shape of typical amorphous ‘halo’ of glasses had significant modifications (i.e., clearly visible 2θ displacements). The pattern of the green sample after NaOH activation actually features some weak peaks consistent with the formation of crystalline N-A-S-H (sodium alumino-silicate hydrated) phases, such as gmelinite (PDF#38-0435), fajausite (PDF#38-0238) and paragonite (PDF#42-0602), although some uncertainties remain, testified by the arrows in Figure 3. Whereas the not perfect match in the position of fajausite (2θ ~ 27°) main peak could be justified by formation of solid solutions, no phase was compatible with the peak at 2θ ~ 39.5°. Interestingly, both gmelinite and fajausite are zeolites [27].

**Figure 4:**
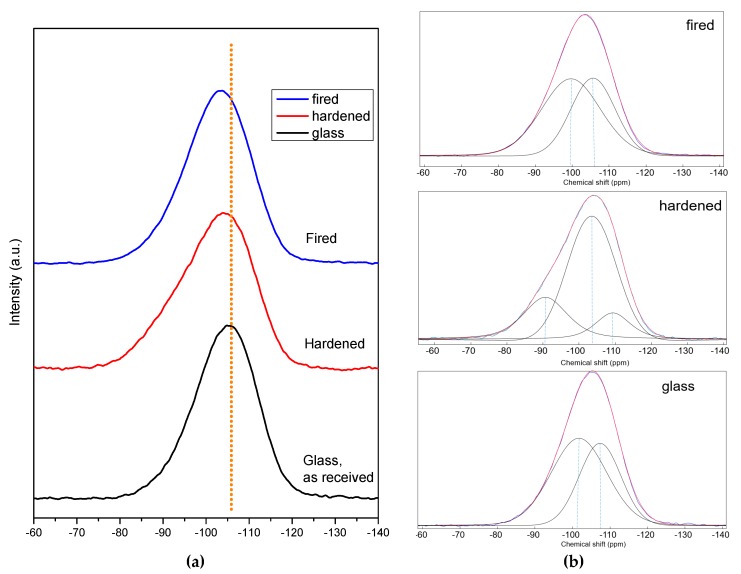
^29^Si NMR studies of BSG glass: (**a**) comparison of spectra in the as received state, after alkali activation (in ‘green’ foams, NaOH/KOH activation) and after firing (in final foams); (**b**) deconvolution analysis: blue lines, from experimental spectra, overlap with fitted curves, in red.

Samples from NaOH/KOH, for which the formation of a zeolitic gel could not be evaluated even as ‘probable’ (from mineralogical analysis), were subjected to NMR studies, which led to interesting observations on the coordination of Si, Al and B ions, caused by the alkali activation and kept in the material after firing. In particular, from the ^29^Si spectra in Figure 4a, we can observe that BSG in the initial state presented a peak centered at about −105 ppm, composed by two equivalent peaks at −107.4 ppm and −101.7 ppm consistent with a mixed glass network (pure silica glass exhibits a peak centered at about −110 ppm, attributed to Q_4_ silicons, SiO_4_ [28,29]). The formation of silanol groups, observed with FTIR in the alkali activated BSG, is confirmed by the study of the deconvoluted broadened spectrum (Figure 4b) that show the presence of three peaks at −109.3, −103.5 and −90.6 ppm. The presence of the peak at higher chemical shift can be attributed to the new hydrated specie SiO_2_(OH)_2_ that is known to provide signals at ca −89 ppm [29]). The quantitative analysis shows that the peak intensity at −109.3 ppm (Q_4_ silicons, SiO_4_) decreases almost the same amount of the peak at −90.6 ppm while the peak at 103.5 remains constant. The peak returned quite symmetrical after firing, which caused dehydration; it is composed by two again equal peaks at −105.63 ppm and −99.66 ppm but with increased upshifting respect the initial state, compared to pure silica glass, attributed to the incorporation of AlO_4_ and BO_4_ units (as observed in geopolymers, the chemical shifts decrease with increasing Al/Si ratio with the formation of Q_4_ (1Al) species [30]).

**Figure 5:**
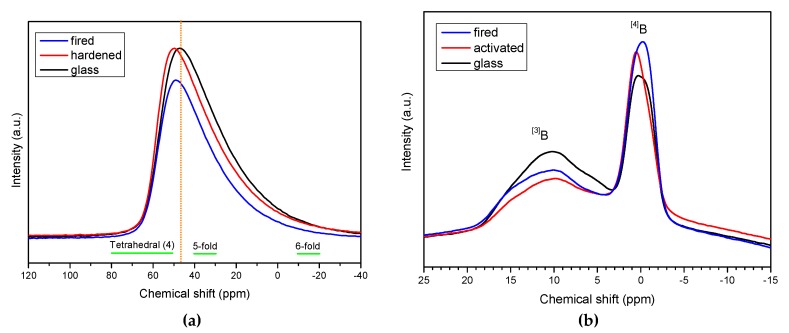
^27^Al (**a**) and ^11^B (**b**) Nuclear magnetic resonance (NMR) studies BSG glass in the as received state, after alkali activation (in ‘green’ foams, NaOH/KOH activation) and after firing (in final foams).

In Figure 5a, the ^27^Al spectra reveal a slight change in the coordination of aluminium ions, passing from the as received state to the hardnened and fired states: the main peak became more symmetrical, at about 47 ppm, likely due to an increase of tetrahedral coordination (signals for 5- and 6-fold co-ordinations are known to prevail at lower chemical shifts [31]).

Finally, a more significant evidence of the structural transformation with alkali activation and firing was provided by ^11^B NMR spectroscopy, as shown in Figure 5b. In BSG, boron oxide evidently acts as network former in a double configuration, forming BO_3_ as well as BO_4_ units, the latter made possible by charge compensation with surrounding alkali ions, like in the case of AlO_4_ units. The two configurations, trigonal and tetrahedral, were maintained with alkali activation and firing but with a different balance. Consistent with the FTIR observations, the tetrahedral coordination was promoted [19]. 

We can posit, at the end, that alkali activation led to a complex amorphous hydrated gel with a boro-alumino-silicate structure (embedding alkali, for charge compensation), amorphous or slightly crystalline; the related spectroscopic signals were weak, due the limited dissolution of the starting glass. The firing treatment, besides causing dehydration, determined an ‘absorption’ of the gel in the molecular structure of BSG: extra Na^+^ and K^+^ ions from the activating solutions were incorporated in the glass structure in the formation of extra BO_4_ and AlO_4_ units.

**Figure 6:**
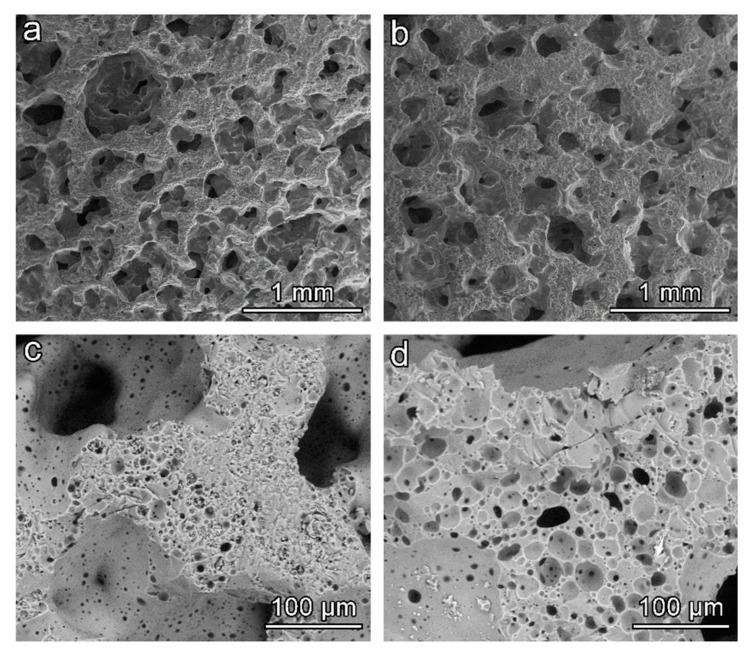
Morphology of BSG-derived glass foams (surfactant: Triton X-100): (**a**,**c**) NaOH activation; (**b**,**d**) NaOH/KOH activation.

The open-celled morphology obtained in the ‘green’ state was confirmed upon firing, for both activating solutions, as shown by Figure 6a,b. The adopted firing temperature was evidently too low for a substantial softening of BSG, 700 °C being the minimum temperature for the sintering of the specific glass (an optimum temperature for glass sintering can be estimated as 50 °C above the dilatometric softening temperature [32], which is 650 °C for BSG [17]). In addition, the limited amount of hydrated phase prevented the foams from a secondary foaming and reshaping of pores (observed for soda-lime glass [5]); the release of water vapour is the likely reason just for small micro-sized pores (darker spots) visible on the surface of struts (Figure 6c,d). 

Table 1 shows that the produced foams exhibited a remarkable compressive strength, considering the open-celled structure. According to the well-known Gibson-Ashby (GA) model [33], the compressive strength of cellular solid, σ_c_, depends on the relative density (ρ_rel_ = 1 − P, where P is the total porosity), as follows:

σ_c_ ≈ σ_bend_ · f(Φ,ρ_rel_) = σ_bend_ · [C·(Φ·ρ_rel_)^3/2^ + (1 − Φ)·ρ_rel_], (1)

where σ_bend_ is the bending strength of the solid phase, C is a dimensionless calibration constant (∼0.2) and f is a ‘structural function’. The quantity (1 − Φ) expresses the mechanical contribution of solid positioned at cell faces, reasonably limited when open porosity is dominant. If we neglect this contribution (Φ ∼ 1), the observed compressive strength could be correlated to a bending strength well exceeding 100 MPa, that is, far above the measured values for pore-free sintered BSG [17].

**Figure 7:**
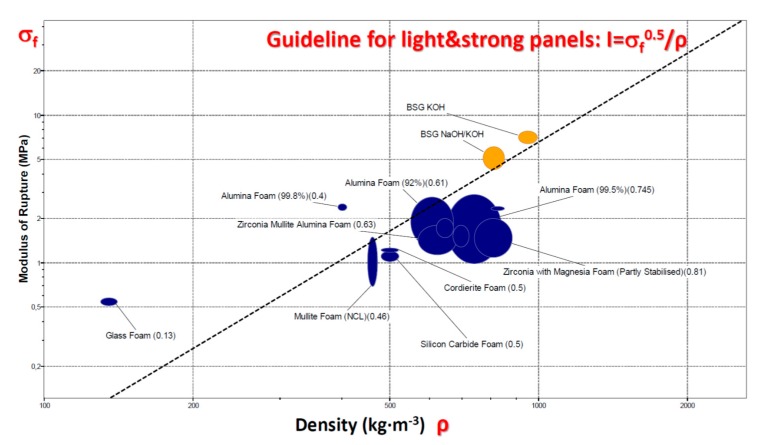
Bending strength/density chart for selected BSG-derived foams compared to commercial ceramic foams.

The favourable strength/density correlation is further testified by the bending strength data illustrated by the Ashby’s plot shown in Figure 7. The plot was traced by means of the CES (Cambridge Engineering Selector, EduPack2017 [34]) and related database concerning ceramic foams. The experimental data from bending strength determinations are highlighted in yellow. Panels, designed to a given applied bending moment, are lighter and lighter with increasing index I, defined as [35]:

I = σ_f_^1/2^/ρ, (2)

where σ_f_ is the real failure stress (bending strength, also known as ‘modulus of rupture’); materials with the same I value stay on the same selection line, in the strength/density chart (see solid line in Figure 7) [35]. The position of newly developed foams in the chart (aligned above the line) makes them good candidate materials for strong lightweight panels, compared to most commercial ceramic foams, with the exception of commercial glass foams (which exploit the mechanical contribution of cell faces, being close-celled [8]). The open-celled morphology could be exploited for the infiltration of a secondary phase (e.g., elastomers, in ballistic protection composites [36]) or for filter manufacturing.

**Figure 8:**
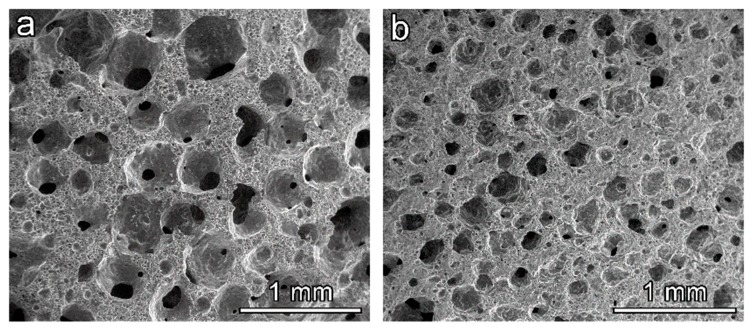
Examples of BSG-derived foams from different surfactants; (**a**) Tween 80; (**b**) SLS.

The proposed approach to cellular glasses is interesting for its inherent flexibility. As evidenced by the experiments with soda-lime glass and bioglass [5,15], the solid content and the duration of both activation and drying stages may affect the viscosity of glass slurries and thus modify the cellular structure in the ‘green’ state. These changes will be the reasonable focus of future investigations, especially at a semi-industrial scale. Figure 8 actually illustrates that the morphology could be tuned even by simply changing the chemistry of the adopted surfactant. The differences in cell size in the derived foams obtained with different surfactants are evident, showing a significant size difference in the macropores detected. Again, despite the open-celled morphology, the crushing strength remained remarkable (see Table 1).

## 4. Conclusions

We may conclude that:

(1) Alkali activation of glass slurries, followed by low temperature sintering, was successfully applied to glass from discarded pharmaceutical vials;
(2) Owing to the specific glass chemistry, the hardening of slurries was not caused by the formation of a C-S-H gel but occurred by formation of a (mostly) amorphous hydrated boro-alumino-silicate gel, in limited quantities;
(3) Alkali activation and subsequent firing determined slight but measurable changes in the molecular structure of the adopted boro-alumino-silicate glass, with enhancement of BO_4_ and AlO_4_ units (by incorporation of ‘activating’ alkali ions);
(4) The cellular structure could be tuned depending on the chemistry of activating solution but also on the chemistry of surfactants used in the foaming of activated glass slurries;
(5) The relatively low firing temperature and the limited quantity of hydrated phases favored the retention of the open-celled morphology, developed upon foaming of activated slurries;
(6) The developed foams, in all processing conditions, exhibited a favorable strength/density correlation.

## Figures and Tables

**Table 1:** Physico-mechanical properties of BSG-derived glass foams produced under different conditions.

Activation	Surfactant	Density (g/cm^3^)	Total Porosity, P (%)	Open Porosity (%)	Compressive Strength (MPa)
NaOH	TritonX-100	0.75 ± 0.01	66 ± 1	55 ± 3	4.3 ± 0.3
NaOH/KOH		0.81 ± 0.02	68 ± 3	52 ± 4	7.7 ± 0.4
NaOH/KOH	Tween 80	0.79 ± 0.01	69 ± 2	55 ± 3	7.2 ± 0.3
NaOH/KOH	SLS	0.98 ± 0.01	61 ± 2	49 ± 2	8.2 ± 0.5
